# High-intensity interval training modulates inflammatory response in Parkinson’s disease

**DOI:** 10.1007/s40520-022-02153-5

**Published:** 2022-06-14

**Authors:** Paulina Malczynska-Sims, Małgorzata Chalimoniuk, Zbigniew Wronski, Jaroslaw Marusiak, Anna Sulek

**Affiliations:** 1 Department of Genetics, Institute of Psychiatry and Neurology, 9 Sobieskiego St, 02-957 Warsaw, Poland; 2 Department of Physical Education and Health in Biała Podlaska, Józef Piłsudski University of Physical Education in Warsaw, 2 Akademicka St, 21-500 Biała Podlaska, Poland; 3 Department of Rehabilitation, Medical Faculty, Medical University of Warsaw, 61 Żwirki i Wigury St., 02-091 Warsaw, Poland; 4 Department of Kinesiology, University School of Physical Education in Wroclaw, Wroclaw, Poland

**Keywords:** Parkinson’s disease, High-intensity interval training, HIIT, Inflammation, Antioxidant capacity

## Abstract

**Background:**

Recent discoveries show that high-intensity interval training (HIIT) can bring many positive effects such as decreases in fat tissue, lower blood sugar levels, improved learning and memory, and lower risk of cardiac disease. Parkinson’s disease (PD) is a neurodegenerative disorder characterized by loss of the dopaminergic neurons, accompanied by chronic inflammation and neuroinflammation. Previous research shows that interval training can bring a beneficial effect on the inflammation and neuroplasticity in PD.

**Objectives:**

The objective of this study was to investigate the effect of 12 weeks of HIIT on the inflammation levels and antioxidant capacity in the serum of PD patients.

**Methods:**

Twenty-eight people diagnosed with PD were enrolled in this study. Fifteen PD patients performed 12 weeks of HIIT on a cycloergometer. Thirteen non-exercised PD patients constitute the control group. Concentrations of inflammation markers and antioxidants’ capacity in the serum were measured at 3 sampling points (a week before, a week after, and 3 months after the HIIT).

**Results:**

Twelve weeks of HIIT decreases the level of TNF-α (*p* = 0.034) and increases the level of IL-10 (*p* = 0.024). Those changes were accompanied by a decreased level of neutrophils (*p* = 0.03), neutrophil/lymphocyte ratio (*p* = 0.048) and neutrophil/monocyte ratio (*p* = 0.0049) with increases in superoxide dismutase levels (*p* = 0.04).

**Conclusions:**

Twelve weeks of HIIT can decrease systemic inflammation in PD patients and improve the antioxidant capacity in their serum, which can slow down the progression of the disease.

## Introduction

Pathological changes in Parkinson’s disease (PD) affect the region in the brain called the substantia nigra pars compacta (SNpc) which is rich in dopaminergic neurons. As a result of many biochemical changes, the death of dopaminergic neurons in this region leads to a decreased level of dopamine in the central nervous system (CNS), and the effect of low dopamine level causes motor disability [1]. Loss of dopamine in the SNpc results in the cascade of further biochemical changes in the neurons such as alpha-synuclein accumulation increased neuroinflammation, and oxidative stress, which leads to neuronal apoptosis [2, 3]. Since all these processes are strictly related, it is difficult to recognize which of the pathological changes is responsible for the disease outbreak. The most probable factor leading to the development of PD is alpha-synuclein accumulation, which involves the activation of microglia and astrocytes, leading to chronic neuroinflammation [4]. During the neuroinflammation processes, the microglia produce many pro-inflammatory cytokines such as tumor necrosis factor-alpha (TNF-α), interleukin 1 beta (IL-1β), interleukin-6 (IL-6) and interferon-gamma (IFN-γ) [2, 5]. During chronic pro-inflammatory microglial activation, an increased level of pro-inflammatory cytokines leads to astrocyte activation and increased production of pro-inflammatory cytokines by those cells. Astrocytes, similarly to the microglia, can produce pro-inflammatory cytokines, and reactive oxygen species (ROS) leading to the cell’s death [2, 5]. Moreover, astrocytes produce two proteins: glial fibrils acidic protein (GFAP) and S100beta protein, which are considered neuroinflammation markers [6]. Under the physiological conditions, increased pro-inflammatory cytokines cause the increased production of anti-inflammatory cytokines such as interleukin-10 (IL-10) or TGF-β, preventing devastating inflammation [7]. In PD, the balance between pro-inflammatory cytokines and anti-inflammatory cytokines is disturbed, causing chronic neuroinflammation. Besides the neuroinflammation, increased brain–blood barrier (BBB) permeability has been observed in PD [2, 8]. Due to the BBB being built from tight junctions among adjacent cells, and besides the five areas in which the permeability is physiologically increased, many substances cannot cross the BBB. As the result of the presence of pro-inflammatory cytokines the BBB can be damaged, which may lead to wider contact between the CNS and peripheral system [8, 9]. Moreover, neuroinflammation, BBB damage, and increased systemic inflammation were reported in PD indicating the close connection between peripheral inflammation and neuroinflammation [10].

The past decade has brought new insights on the importance of physical activity in PD. Exercise programs for people with PD mostly consider aerobic and resistance trainings, though recent years have shed a new light on the importance of interval training in a PD rehabilitation program [11–13, 48, 49]. Research shows that interval training leads to an increased BDNF level and has an influence on the inflammation in PD [11–13]. Studies show that besides the biochemical changes after that occur after interval training in PD, such exercise can influence on the UPDRS scale and can even lead to the slowing down of the progression of the disease [12, 13]. However, many researchers have shown that high-intensity exercise might have a pro-inflammatory effect, and that long-term high-intensity interval training (HIIT) might bring more benefits in the basal level of the pro-inflammatory factors rather than this happening after a single session [11, 14].

In this research, we investigate the influence of 12 weeks of HIIT on inflammation markers and antioxidant capacity in the serum of PD patients. The research had the following detailed aims: to investigate whether the 12 weeks of HIIT influenced a) the level of TNF-α, IL-1β, IL-6, IL-10, and leukocytes, as inflammation markers in the serum of PD patients; b) GFAP and S100beta levels as the neuroinflammation markers in the serum of PD patients; c) superoxide dismutase (SOD), and total glutathione (GSH) level and catalase (CAT) as antioxidant capacity markers in the serum of PD patients. The authors hypothesized that 12 weeks of HIIT had a positive effect on the inflammation and neuroinflammation markers accompanied by improved antioxidant capacity.

## Materials and methods

### Participants

Twenty-eight people with diagnosed Parkinson’s disease were enrolled in the study. All participants had to meet the following criteria: be a person with diagnosed PD at an early-to-intermittent stage of the disease according to the H&Y scale (1–2.5), be taking antiparkinson medication, have no serious cardiac disease, psychiatric disorders, or other neurological diseases, and be able to perform 12 weeks of high-intensity interval training. Participants were non-randomly assigned to two groups: 1) The high-intensity interval group (TR-PD), and 2) the non-exercise control group (NTR-PD). Fifteen participants agreed to perform 12 weeks of HIIT (9 men and 6 women). Thirteen participants agreed to be a control group (6 men and 7 women). The demographic and clinical features of both groups are summarized in Table 1. The minimal sample size group was calculated before the study. It allowed to conclude that at least 15 people for each group had to take part in this research to obtain statistically significant results (with a margin error of 25%, and a confidence level of 95%). In the TR-PD group, there were no drop-outs due to COVID-19 or any other reason. All participants from this group attended all measuring points. In the NTR-PD group, two participants did not attend the measurements in T_2_ sampling points for other reasons. This study was approved by the Bioethical Commission of the Institute of Psychiatry and Neurology in Warsaw and carried out in accordance with the principles of the Declaration of Helsinki. All participants were informed about the study and provided written informed consent.

**Table 1:** The clinical and demographical features of participants

	TR-PD (*n* = 15)	NTR-PD (*n* = 13)	*p* value
Age	64.24 ± 1.4	67.0 ± 2.4	0.29
Gender (M/F)	9/6	6/7	0.46
Body height (cm)	168.3 ± 2.1	168.0 ± 2.7	0.76
Body mass (kg)	75.4 ± 3.9	70.31 ± 4.2	0.3
BMI	26.55 ± 1.3	24.87 ± 1.45	0.17
H&Y scale	1.93 ± 0.12	2.04 ± 0.07	0.57
UPDRS	36.36 ± 4.87	41.73 ± 3.53	0.26
UPDRS part III	26.18 ± 3.67	28.96 ± 2.37	0.22
Disease duration	5.8 ± 1.1	8.2 ± 1.04	0.1
L-dopa intake (mg)	572.1 ± 107.6	628.3 ± 102.3	0.6

*BMI* body mass index, *H&Y scale* Hoehn and Yahr scale, *UPDRS* Unified Parkinson’s Disease Rating Scale, *TR-PD* training group, *NTR-PD* control group

Values are presented as mean ± SEM

### Study design

**Fig. 1:**
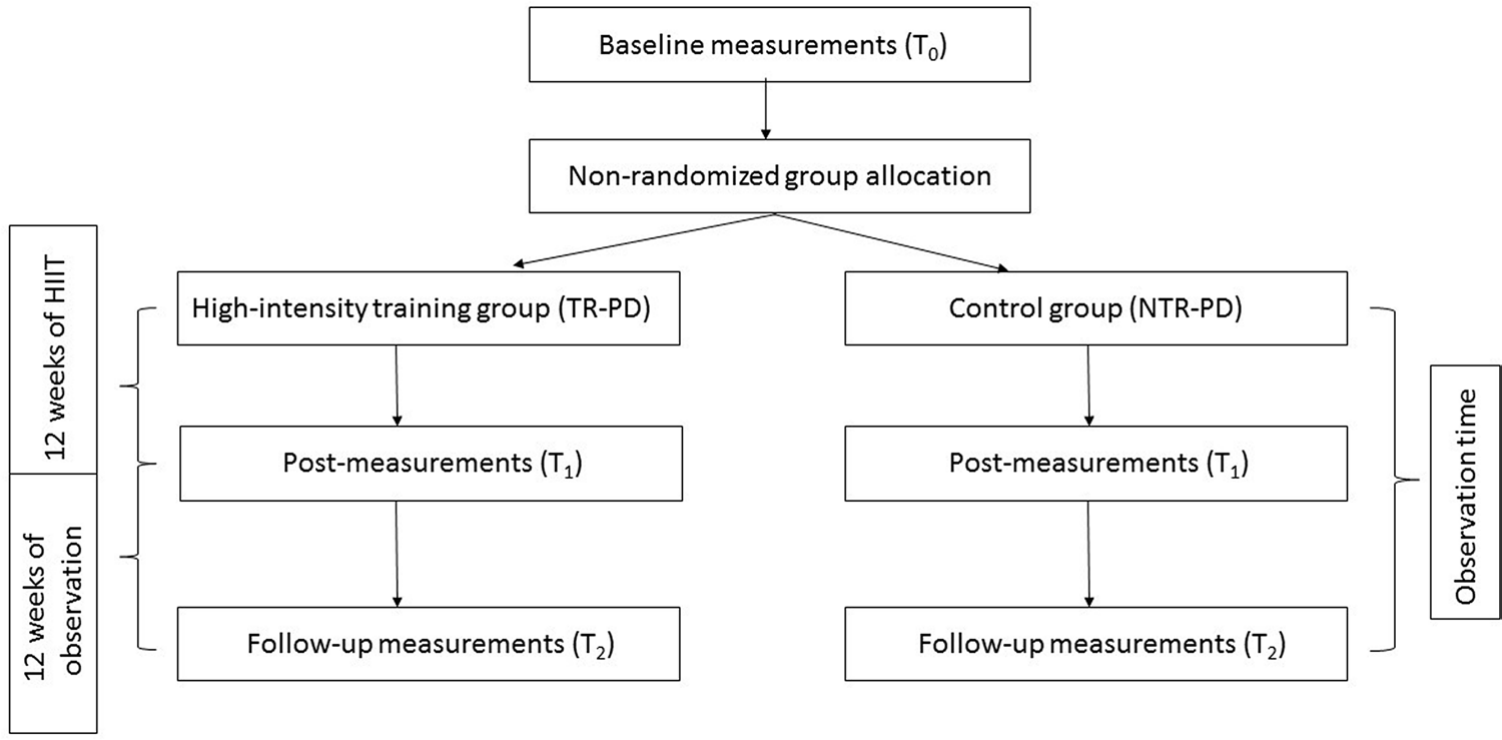
The general scheme of the study design

One week before the 12 weeks of HIIT (T_0_), participants from both groups were examined by a neurologist who determined the disease stage using the Hoehn and Yahr scale (H&Y scale) [15] and the Unified Parkinson’s Disease Scale (UPDRS) [16]. All participants were in their OFF stage (without the medication). The last anti-parkinson medication was taken a day earlier at 8:00 pm. During the neurological examination blood samples were taken from all participants. All patients fasted for 12 h before the samples were taken. A week after the intervention ended, blood samples were taken once more from all of the participants, and again after the 12 h of fasting (post-measurements, T_1_). To determine the duration of blood changes, 12 weeks after the HIIT ended (3 months / 12 weeks training) blood samples were taken from all participants (Follow-up measurements, T_2_). Blood samples were taken in the morning after 12 h of fasting. The study design is presented in Fig. 1.

### High-intensity interval training protocol

Fifteen people (the TR-PD group) performed 12-week of HIIT. The training cycle was similar to that effectively applied previously in PD patients [12, 13]. The difference between two cycles was their duration (12 weeks). In each week patients had to perform 3 exercise-training sessions on a stationary cycloergometer (MONARK, Sweden) for a total of 36 sessions. This HIIT cycle consisted of three 1-h training sessions performed three times per week, giving a total of 36 training sessions during the 12-week cycle. In each training session, the exercise part consisted of 10 sets of 4 min of interval training, including 2-min cycling at ≥ 60 (rpm), but preferably at 80–90 (rpm) (fast phase of an interval) and 2-min cycling at ≤ 60 (rpm) (slow phase of an interval). The training, performed on a stationary cycloergometer, allowed us to measure cadence (rpm) and power [W]. The heart rate (HR, bpm) was measured by the Polar system (Polar, Finland). The training supervisor adjusted the resistance for each patient to ensure they cycled at their each target heart rate (THR) and appropriate speed. All training sessions were performed during the patients’ medication on-phase (beneficial effect of anti-parkinson medication). The patients were exercised on the cycloergometer at 60–80% of their individualized HRmax (HR max calculation using the Karvonen–Tanaka formula) [17]. During the fast phase of the interval training patients were encouraged to cycle faster (80–90 rpm, or 30% faster) than their preferred voluntary speed. Each patient had his/her target heart rate increased every 2 weeks by 5%, from 60% up to 80% in the following order: 60% of the HRmax during the first two weeks, 65% during 3–4 weeks, 70% during 5–6 weeks, 75% during 7–9 weeks, and 80% during 10–12 weeks of the training period. The training supervisor provided water and any additional help during the training session.

### Detection of pro-inflammatory factors and antioxidants

Blood was collected into 2 tubes from all of the participants after the 12 h of fasting in the morning hours. In one tube with EDTA, blood for the determination of white blood cells counts was collected. Blood from the second tube, without an anticoagulant was centrifuged at 3000 rpm at 4 °C per 10 min to obtain serum. Further, serum was replaced into 500 µL tubes (400 µL of serum in each tube) and stored at -80 °C for further use. Serum cytokine levels were measured using the sandwich ELISA method. TNF-α was measured by Quantikine TNF-α kit (R&D Systems, Minneapolis, USA). Levels of IL-1β, IL-6, IL-10, GFAP, and S100beta were determined by DuoSets kits (R&D Systems, Minneapolis, USA) according to the manufacturer's procedure. The total count and percentage of leukocytes, neutrophils, lymphocytes, and monocytes were determined on the Pentra DX device, Nexus (Horiba). Levels of antioxidants (GSH, SOD, and CAT) in the serum were measured by colorimetric kit (ThermoFisher, Massachusetts, USA) according to the manufacturer’s protocol. Determination of leukocyte count allowed the calculation of ratios between hematological parameters. The neutrophil to lymphocyte ratio (NLR) was calculated by dividing the count of neutrophils by the count of monocytes; the neutrophil to monocyte ratio (NMR) was calculated by dividing the count of neutrophils by the count of monocytes; the systemic immune-inflammation index (SII) was calculated by multiplication of NLR by platelet count; and the monocyte to lymphocyte ratio (MLR) was calculated by dividing the count of monocytes by the count of lymphocytes.

## Statistical analysis

All statistical analyses were performed using the GraphPad Prism 5 (San Diego, California, USA). Non-parametric *U* Mann–Whitney test was performed for the determination between groups at each point. Non-parametric Wilcoxon test was used to determine the changes between sampling points in groups. All data were earlier screened for missing data and outliers, which were identified as Q1-(1.5*IQR) or Q3 + (1.5*IQR). Outliers were removed from the further analysis. Statistical significance was established at *p* ≤ 0.05.

## Results

### Inflammation

Table 2 displays the change in the concentration of TNF-α, IL-1β, IL-6, IL-10, GFAP, and S100beta in both groups in 3 measurement points. In the TR-PD group, TNF-α concentration decreased after the HIIT program. 12 weeks after the HIIT ended the level of TNF-α in the training group increased comparing T_1_ and T_2_ points; however, this change was not statistical. There was no change in TNF-α concentration in the NTR-PD group.

**Table 2:** Changes in the cytokines’ levels in NTR-PD and PD-TR group

Variables	TR-PD					NTR-PD							
	T_0_	T_1_	T_2_	*p*^1^	*p*^2^	T_0_	T_1_	T_2_	*p*^3^	*p*^4^	*p*^5^	*p*^6^	*p*^7^
TNF-α (pg/mL)	12.96 ± 0.84 (13.04)	10.09 ± 1.04 (10.54)	12.22 ± 1.6 (12.13)	0.034^a^	0.62	16.26 ± 2.68 (12)	16.55 ± 3.67 (11.47)	14.58 ± 1.95 (13.59)	0.46	0.64	0.6	0.6	0.43
IL-1β (pg/mL)	0.14 ± 0.09 (0)	0.15 ± 0.1 (0)	1.1 ± 0.43 (0.77)	1.0	0.15	0.72 ± 0.27 (0.6)	0.43 ± 0.43 (0)	1.2 ± 0.65 (0.32)	0.4	0.12	0.052	0.86	0.77
IL-6 (pg/mL)	14.03 ± 6.2 (1.64)	4.23 ± 2.01 (0.2)	5.78 ± 2.6 (1.46)	0.47	0.3	9.37 ± 6.36 (0.16)	9.45 ± 6.14 (3.72)	21.06 ± 12.31 (3.87)	0.36	0.92	0.7	0.1	0.46
IL-10 (pg/mL)	1.4 ± 0.4 (0.83)	44.11 ± 22.38 (5.2)	15.75 ± 7.04 (6.4)	0.023^a^	0.2	16.10 ± 14.31 (1.2)	18.92 ± 10.47 (4.74)	6.06 ± 1.6 (6.43)	0.8	0.84	0.68	0.8	0.64
IL-10/TNF-α ratio	1.38 ± 1.23 (0.1)	3.88 ± 1.9 (0.68)	2.45 ± 0.97 (0.93)	0.15	1.0	1.03 ± 0.72 (0.39)	1.36 ± 0.76 (0.35)	0.58 ± 0.16 (0.62)	1.0	1.0	0.5	0.25	0.3
GFAP (pg/mL)	0.23 ± 0.03 (0.03)	0.25 ± 0.09 (0.067)	0.26 ± 0.09 (0.11)	0.67	0.57	0.35 ± 0.22 (0.12)	0.27 ± 0.08 (0.22)	0.94 ± 0.5 (0.24)	0.2	0.8	0.54	0.6	0.5
S100beta (pg/mL)	105.6 ± 22.66 (78.76)	227.8 ± 41.14 (188.3)	213.5 ± 44.12 (173.1)	0.0017^a^	0.78	96.81 ± 23.10 (98.85)	165 ± 24.62 (179.9)	168.5 ± 37.45 (119)	0.024^a^	0.4	0.96	0.45	0.55

*TNF-α* tumor necrosis factor alpha, *IL-1β* interleukin 1 beta, *IL-6* interleukin 6, *IL-10* interleukin 10, *IL-10/TNF-α ratio* interleukin 10 to tumor necrosis factor alpha ratio, *GFAP* glial fibrils acidic protein, *S100beta* calcium binding protein S100 beta, *SOD* sodium dismutase, *GSH* glutathione, *CAT* catalase, *TR-PD* training group, *NTR-PD* control group

*p*^*1*^ *p* value for the Wilcoxon in-group comparison for TR-PD group between T_0 _and T_1 _sampling points

*p*^*2*^ *p* value for the Wilcoxon in-group comparison for TR-PD group between T_1 _and T_2 _sampling points

*p*^*3*^ *p* value for the Wilcoxon in-group comparison for NTR-PD group between T_0 _and T_1 _sampling points

*p*^*4*^ *p* value for the Wilcoxon in-group comparison for NTR-PD group between T_1 _and T_2_ sampling points

*p*^*5*^ *p* value for the Mann–Whitney between-group comparison between TR-PD and NTR-PD at T_0_ sampling points

*p*^*6*^ *p* value for the Mann–Whitney between-group comparison between TR-PD and NTR-PD at T_1_ sampling points

*p*^*7*^ *p* value for the Mann–Whitney between-group comparison between TR-PD and NTR-PD at T_2_ sampling points

Values are presented as mean ± SEM (median)

^a^statistical significance (*p* < 0.05) for the Wilcoxon in-group comparison between T_0 _and T_1 _sampling points

^b^statistical significance (*p* < 0.05) for the Wilcoxon in-group comparison between T_1_ and T_2_ sampling points

^c^statistical significance (*p* < 0.05) for the Mann–Whitney between-group comparison

No changes after the HIIT were observed in the TR-PD group compared to the baseline level of IL-1β and 3 months after the HIIT. No changes in the NTR-PD group were observed during the experiment (Table 2).

No statistical changes in the concentration of the IL-6 were determined in the TR-PD group and in the NTR-PD group during the study (Table 2). In the TR-PD group level of this interleukin decreases after the HIIT; however, this is not statistically significant. In the NTR-PD group, no changes in the IL-6 concentration were observed.

Twelve weeks of HIIT increased the level of IL-10 in the TR-PD group. No changes in the NTR-PD group were observed during the study (Table 2).

Twelve weeks of HIIT did not change the IL-10/TNF-α ratio in the TR-PD group neither after 3 months. Nor were any changes observed in the NTR-PD group (Table 2).

Twelve weeks of HIIT did not affect the GFAP level in the serum of the PD patients (Table 2) who performed the training and 3 months after the training completion. The GFAP level did not change in the control group during the observation period.

The level of S100beta protein in the serum increased statistically after the 12 weeks of HIIT in the TR-PD group (Table 2) and did not change within 3 months after the training. In the control group, the increased level of S100beta was observed during the first three months of observation, without any changes during the last 3 months of observation.

### White blood cells

Table 3 presents changes in the leukocyte count in both groups during the 6 months of observations. No changes were observed in the TR-PD group after 12 weeks of HIIT and 3 months after the training was completed. No changes were observed in the NTR-PD group during the first three months of observation and the last 3 months of observation.

**Table 3:** Changes in the hematological parameters and the immune inflammatory markers in NTR-PD and TR-PD groups

Variables	TR-PD					NTR-PD							
	T_0_	T_1_	T_2_	*p*^1^	*p*^2^	T_0_	T_1_	T_2_	*p*^3^	*p*^4^	*p*^5^	*p*^6^	*p*^7^
Leukocytes (K/u)	6.14 ± 0.48 (5.76)	5.69 ± 0.24 (5.55)	5.56 ± 0.24 (5.5)	0.24	0.22	5.87 ± 0.3 (5.9)	6.11 ± 0.4 (6.1)	5.86 ± 0.32 (5.7)	0.67	0.38	0.68	0.38	0.37
Neutrophils (K/u)	4.27 ± 0.6 (3.68)	3.3 ± 0.23 (3.1)	3.06 ± 0.2 (3.14)	0.03^a^	0.53	3.25 ± 0.27 (3.18)	3.14 ± 0.26 (2.87)	3.12 ± 0.25 (3.0)	0.27	0.53	0.38	0.55	0.78
Lymphocytes (K/u)	1.44 ± 0.08 (1.5)	1.73 ± 0.12 (1.72)	1.78 ± 0.12 (1.65)	0.12	0.9	1.9 ± 0.19 (1.9)	1.96 ± 0.09 (1.97)	1.93 ± 0.11 (2.06)	0.32	0.43	0.12	0.12	0.34
Monocytes (K/u)	0.45 ± 0.03 (0.45)	0.56 ± 0.05 (0.56)	0.48 ± 0.016 (0.46)	0.07	0.09	0.45 ± 0.06 (0.36)	0.6 ± 0.07 (0.59)	0.58 ± 0.05 (0.51)	0.0005^a^	0.78	0.08	0.08	0.08
NLR	2.58 ± 0.3 (2.66)	1.8 ± 0.13 (1.75)	1.83 ± 0.18 (1.66)	0.048^a^	0.54	1.68 ± 0.17 (1.61)	1.57 ± 0.13 (1.6)	1.6 ± 0.16 (1.49)	0.2	0.9	0.08	0.32	0.3
NMR	8.3 ± 0.8 (8.36)	5.21 ± 0.34 (5.9)	6.23 ± 0.6 (6.05)	0.0049^a^	0.13	7.76 ± 0.8 (6.5)	5.7 ± 0.52 (5.62)	5.8 ± 0.6 (5.54)	0.001^a^	0.94	0.5	0.54	0.52
MLR	0.29 ± 0.02 (0.28)	0.32 ± 0.02 (0.3)	0.29 ± 0.012 (0.3)	0.19	0.24	0.23 ± 0.02 (0.23)	0.29 ± 0.03 (0.29)	0.3 ± 0.025 (0.28)	0.01^a^	0.85	0.06	0.4	0.9
SII	632.2 ± 88.01 (666.7)	475.2 ± 40.92 (411)^a^	428 ± 39.11 (509.4)	0.035	0.19	368.3 ± 47.07 (330.7)^b^	392.1 ± 46.28 (352.8)	396.4 ± 56.85 (326.3)	1.0	0.96	0.03^b^	0.13	0.57

*NLR* neutrophil to lymphocyte ratio, *NMR* neutrophil to monocyte ratio, *MLR* monocyte to lymphocyte ratio, *SII* systemic-immune inflammatory index

Values are presented as mean ± SEM (median)

*p*^*1*^ *p* value for the Wilcoxon in-group comparison for TR-PD group between T_0 _and T_1 _sampling points

*p*^*2*^ *p* value for the Wilcoxon in-group comparison for TR-PD group between T_1 _and T_2 _sampling points

*p*^*3*^ *p* value for the Wilcoxon in-group comparison for NTR-PD group between T_0 _and T_1 _sampling points

*p*^*4*^ *p* value for the Wilcoxon in-group comparison for NTR-PD group between T_1 _and T_2_ sampling points

*p*^*5*^ *p* value for the Mann–Whitney between-group comparison between TR-PD and NTR-PD at T_0_ sampling points

*p*^*6*^ *p* value for the Mann–Whitney between-group comparison between TR-PD and NTR-PD at T_1_ sampling points

*p*^*7*^ *p* value for the Mann–Whitney between-group comparison between TR-PD and NTR-PD at T_2_ sampling points

^a^statistical significance (*p* < 0.05) for the Wilcoxon in-group comparison between T_0 _and T_1 _sampling points

^b^statistical significance (*p* < 0.05) for the Mann–Whitney between-group comparison

In this research, HIIT was found to decrease the count of neutrophils in the TR-PD group (Table 3), with no changes during the 3 months after the training was completed. In the control group, the count of neutrophils did not change during the observation.

HIIT did not affect the count of lymphocytes in the TR-PD group. Its level did not change 3 months after the HIIT. Similarly to the TR-PD group in the NTR-PD group, no changes in the count of lymphocytes were observed after the first 3 months and at the end of the observation period.

The level of monocyte shows a tendency to increase after the HIIT in the TR-PD group; however, this change was not statistically significant. In the NTR-PD group the level of monocytes increased statistically at the T_1_ sampling point compared to the basal level. No changes were observed in both groups 3 months after HIIT.

### Cellular immune inflammation markers

Table 3 presents changes in the level of inflammatory indexes during the study in both groups.

In this study, the neutrophil/lymphocyte ratio level decreased after the 12 weeks of HIIT in the TR-PD group with no changes in the NTR-PD group. At the T_2_ sampling point, no changes in the neutrophil/lymphocyte ratio levels were observed in either the TR-PD or NTR-PD groups. A higher neutrophil/lymphocyte ratio was observed in TR-PD patients at the starting point T_0_.

The neutrophil/monocyte ratio level statistically decreased after the HIIT in the TR-PD group and stayed at the same level 3 months after HIIT. In the NTR-PD group, the lower level of neutrophil/monocyte ratio was observed after 3 months with no changes within the next 3 months.

Analysis of the monocyte/lymphocyte ratio showed that HIIT did not affect its level in the TR-PD group, with the increase in the NTR-PD group at the same time. The level of monocyte/lymphocyte ratio did not change 3 months after the HIIT was completed in the TR-PD group nor in the NTR-PD group.

The systemic immune-inflammation index level statistically decreased after the HIIT in the TR-PD group, with no changes between T_1_ and T_2_ sampling points. In the NTR-PD group, no changes between T_0_ and T_1_ sampling points were observed as well as between the T_1_ and T_2_ sampling points. At the T_0_ sampling point, SII was statistically higher in the TR-PD group, with no differences between groups at the T_1_ and the T_2_ sampling points.

### Antioxidants

Table 4 displays changes in the antioxidants level in the TR-PD and NTR-PD groups at three sampling points. No statistical changes between the T_0_ and T_1_ points were reported in the TR-PD group nor in the NTR-PD group in the level of total GSH in the serum. No statistical changes were observed at the T_2_ sampling point in both groups.

**Table 4:** Changes in antioxidants capacity in NTR-PD and TR-PD groups

Variables	TR-PD					NTR-PD							
	T_0_	T_1_	T_2_	*p*^1^	*p*^2^	T_0_	T_1_	T_2_	*p*^3^	*p*^4^	*p*^5^	*p*^6^	*p*^7^
SOD	7.45 ± 0.55 (7.76)	8.92 ± 0.5 (8.3)	7.7 ± 0.7 (7.4)	0.04^a^	0.008^b^	5.87 ± 0.4 (5.82)	5.75 ± 0.53 (5.53)	6.29 ± 0.42 (6.2)	0.64	0.56	0.05^c^	0.0008^c^	0.2
Total GSH	4.088 ± 0.5 (3.9)	4.56 ± 0.8 (3.5)	4.38 ± 0.6 (3.9)	0.3	0.67	3.15 ± 0.46 (2.47)	3.56 ± 0.4 (3.65)	5.7 ± 0.9 (5.7)	0.16	0.09	0.25	0.8	0.16
CAT	17.12 ± 1.55 (17.65)	19.97 ± 0.9 (21.29)	22.24 ± 0.58 (23.03)	0.16	0.22	19.08 ± 0.49 (19.35)	19.01 ± 0.97 (19.10)	21.43 ± 0.75 (21.52)	0.57	0.1	0.14	0.5	0.43

*SOD* sodium dismutase, *GSH* glutathione, *CAT* catalase, *TR-PD* training group, *NTR-PD* control group

*p*^*1*^ *p* value for the Wilcoxon in-group comparison for TR-PD group between T_0 _and T_1 _sampling points

*p*^*2*^ *p* value for the Wilcoxon in-group comparison for TR-PD group between T_1 _and T_2 _sampling points

p^3^*p* value for the Wilcoxon in-group comparison for NTR-PD group between T_0 _and T_1 _sampling points

*p*^*4*^ *p* value for the Wilcoxon in-group comparison for NTR-PD group between T_1 _and T_2_ sampling points

*p*^*5*^ *p* value for the Mann–Whitney between-group comparison between TR-PD and NTR-PD at T_0_ sampling points

*p*^*6*^ *p* value for the Mann–Whitney between-group comparison between TR-PD and NTR-PD at T_1_ sampling points

*p*^*7*^ *p* value for the Mann–Whitney between-group comparison between TR-PD and NTR-PD at T_2_ sampling points

Values are presented as mean ± SEM (median)

^a^statistical significance (*p* < 0.05) for the Wilcoxon in-group comparison between T_0 _and T_1 _sampling points

^b^statistical significance (*p* < 0.05) for the Wilcoxon in-group comparison between T_1_ and T_2_ sampling points

^c^statistical significance (*p* < 0.05) for the Mann–Whitney between-group comparison

The serum level of SOD increased after the HIIT in the TR-PD group and decreased to the basal level at the T_2_ sampling point. In the NTR-PD group, no changes in SOD activity were observed during the study. SOD activity was higher in the TR-PD group compared to the NTR-PD group at the starting point and at the T_1_ point but not at the T_2_ sampling point.

The CAT levels in serum did not change in the TR-PD group, nor in the NTR-PD group during the study.

### Discussion

In this study, we observed that 12 weeks of HIIT performed 3 times per week for 60 min (each session involving cycling on a cycloergometer) improved inflammation and antioxidant capacity.

### Changes in inflammation status

Twelve weeks of HIIT decreased the level of TNF-α in TR-PD, which is in agreement with studies conducted by Zoladz et al. [13] and Szymura et al. [18]. The only difference between the first study of these studies and this one is the fact that in our study PD patients performed HIIT, while in the previous study researchers decided to perform moderate-intensity interval training. The same observation was made by Szymura et al. [18] after 12 weeks of balance training in PD, which decreased the level of TNF-α. Only Landers et al. [19] showed no statistical changes in the TNF-α before and after the training. In this study, a decrease of IL-6 concentration was observed; however, those changes were not statistically significant due to too high SEM. No changes between pre-and post-measurements in IL-6 were observed by Landers et al. [19]; however, in their study, the level was stable without the tendency to decrease as was the case in our study. It is important to highlight that the timing of the blood collection method was different in both studies: Landers et al. [19] collected samples between 48 and 72 h after the physical activity, in this study, blood was collected a week after the training completion, to avoid short changes in interleukin levels caused by a single session of HIIT rather than the whole program. The same observations was made by Szymura et al. [18], in whose study 12 weeks of balance training did not affect the level of IL-6. Besides changes in levels of those two cytokines, the elevated level of IL-10 was found in this study. A similar observation was made by Landers et al. [19] and Szymura et al. [18]. It is important to highlight the point that IL-6 induces the production of IL-10 [20]. Moreover, muscle contraction during the exercise induces the production of IL-6, which leads to the elevation of the level of IL-10 [21]. Elevation of IL-6 was not observed in this study, probably due to the long period between the training and blood sampling; however, the elevation of IL-10 was observed. IL-10 is an important cytokine, which has anti-inflammation purposes, helping in tissue healing and regeneration [22]. Its elevated concentration is desired in the case of PD due to its potential role in silencing inflammation. No changes in the concentration of IL-1β after the HIIT were observed in this study. Its level stayed low, being limited to increase after the recovery time. The IL-10/TNF-α ratio average concentration increased after the HIIT, but this was not significant due to there being no changes in the median concentration. Our results are different from those presented by Landers et al. [19], who observed the statistically increased level of this ratio. These differences might be due to the two different statistical tests used. In this research, to investigate the change between the T_0_ and T_1_ points, the non-parametric Wilcoxon test was used, which compares the median rather than average concentrations and is suitable for the comparison of in-group changes, while Landers et al. [19] used a non-parametric Mann–Whitney test which is more likely to be used for in-between group comparisons. It is important to highlight the point that during the observation time no changes in any of the cytokines were observed in the control group.

### Neuroinflammation markers

To our knowledge, this is the first study that shows the concentration of two neuroinflammation markers in PD in the context of the influence of HIIT. In both groups, there were no changes in the GFAP level across period of the study. It seems that the GFAP was not willing to change its level even after the HIIT. What is interesting is that S100beta increased in both groups, with a bigger change in the TR-PD group, and showed a decreasing tendency 12 weeks after the HIIT in the TR-PD group. Similar observations were reported by Battista et al. [23] in research focused on young adults. While the GFAP level did not differ after HIIT in young adults, the level of S100beta increased as the result of training [23]. Moreover, in the animal model of PD, after prolonged training the levels of GFAP and S100beta were decreased in the brain [24]. Previous research showed that S100beta increases after aerobic exercise [25–27]; however, other studies have shown that endurance exercises did not change the level of S100beta [28, 29]. Moreover, it was suggested that S100beta can be released from the skeletal muscle as the response to the muscle damage caused by intensive exercise [25, 27]. In this study we observed an increased level of S00beta after HIIT in the TR-PD group and after 3 first months of observation in the NTR-PD group. In TR-PD, the increase of S100beta concentration of about 115% after HIIT was observed. This increase was significant, suggesting the source of S100beta in the TR-PD group might not only have been the brain, but that this protein might be released from the muscles as the result of the muscle damage during exertion. We are assuming that the increase of S100beta level of about 70% in the NTR-PD could be a physiological increase connected with the progression of the disease. Additionally, the tendency of the S100beta level to decrease at the T_2_ point in the TR-PD group, and increase at the same sampling point in the NTR-PD group, suggested that the increase in the S100beta level in TR-PD was more likely connected to HIIT rather than to disease progression and might have a dual source.

### White blood cells

To our knowledge, this is the first research to investigates the influence of HIIT on white blood cells in PD patients. After the HIIT the significant decrease of neutrophils in the TR-PD group was noted, while in the NTR-PD group no changes in the count of neutrophils were observed. In the young population, researchers showed that prolonged training did not affect the neutrophils count in a healthy population [30, 31], nor the elderly people with rheumatoid arthritis [32]. This effect seems to be the opposite in a healthy population compared to the population affected with chronic inflammation, showing a beneficial effect of prolonged training in this second group. In this study, no changes in the leukocytes count were observed in both groups, which is in agreement with studies showing that prolonged training did not affect the count of leukocytes in young adults [30, 31, 33] and elderly people with rheumatoid arthritis [32]. No changes in the lymphocytes count after HIIT confirmed studies in the young population [30, 31] as well as in elderly people with rheumatoid arthritis [32]. What is more interesting in this research is that the count of monocytes was elevated at the T_1_ sampling point in the NTR-PD, group with no significant elevation in the TR-PD group. This study is similar to other studies showing no changes in the monocytes count as the result of prolonged training in young adults [30, 31] and the elderly population with rheumatoid arthritis [32].

### Cellular immune inflammation markers

Our research showed that after 12 weeks of HIIT the level of three cellular immune inflammation markers decreased in the PD-TR group, while in the control group, the decrease in the neutrophil/monocyte ratio and increase in the monocyte/lymphocyte ratio were observed. The neutrophil/lymphocyte ratio is considered a good marker of inflammation and was previously described as elevated in PD patients [34–37]. In the context of physical exercise, its level was reported elevated after the exercise and decreased after the long-term training events [38]. In this research the decreased level of the neutrophil/lymphocyte ratio was found as the result of HIIT, confirming observations from other studies [39–41]. A similar observation was a neutrophil/monocyte ratio level in both groups, indicating that those changes might not be connected with the HIIT intervention. The systemic immune-inflammation index level, similar to the neutrophil/lymphocyte ratio, was found to increase after a single session of exercise and decrease after the long-term training [39, 42]. In this research, the decreased level of the systemic immune-inflammation index after the HIIT was found, with no changes in the NTR-PD group. These findings confirmed that HIIT can decrease systemic inflammation in PD patients and can be a good supportive non-pharmacological treatment for this neurodegenerative disease.

### Antioxidant capacity

Antioxidants play an important role in ROS neutralization and cell protection. They can be divided into two groups such as protein and non-protein defense systems. In the first group are two antioxidants analyzed in this research. SOD is one of the most important antioxidants, placed in mitochondria, and the main source of ROS in the cells. It has two isoforms, SOD1 and SOD2, which are placed in different parts of mitochondria [43]. In this research the SOD level statistically increased after 12 weeks of HIIT in the TR-PD group with no changes in the NTR-PD group. Landers et al. [19] presented that the SOD1 level was not changed after the high-intensity multimodal exercise boot camp, which is not in accordance with this study. This increase could be explained by the fact that during exercise muscles produce higher amounts of ROS. Due to the increase in oxidative stress, a higher need for SOD activity is understandable [44].

CAT is an enzyme that takes part in ROS neutralization. Its decreased level was previously reported in PD and can be one of the mechanisms responsible for the lower ROS defense [45]. In this research, no statistical changes in CAT level after HIIT were reported; however, its level tended to increase within the study in the TR-PD group, and between the T_1_ and T_2_ points in the NTR-PD group. It is interesting that at the T_2_ point, in general, inflammation status and antioxidant capacity in the NTR-PD group improved, suggesting the involvement of unknown factors in the improvement in the NTR-PD group. Studies show that CAT levels increased after the HIIT in young adults after the exercise and returns to the baseline within 48 h [46]. These findings can explain the fact that after 1 week of the HIIT, the level of CAT was not statistically increased in the TR-PD group; however, it is hard to explain why it is still increased at the T_2_ point.

Total GSH in the serum showed no changes in its level after HIIT in the TR-PD group. During the study no changes in total GSH were reported in the NTR-PD group either. In contrast, in young adults after HIIT the blood level of GSH increased when compared to the basal level [47]. In this research, the focus was on the total glutathione level as a non-enzymatic mechanism of ROS defense. The purpose of that was to investigate whether HIIT can affect not only the enzymatic part of antioxidants but also the non-enzymatic defense as the importance of the ROS neutralization mechanism.

### Limitations

One of the limitations of this study was the sample size. Although the minimal sample size was calculated, the number of participants taking part in the study was not satisfactory. Moreover, due to the relatively short period of training and rest time, the neurological examination after the HIIT may not show sufficiently significant effects.

## Conclusions

This research showed that 12 weeks of HIIT can decrease the level of pro-inflammatory cytokines and improve the antioxidant capacity. However, some of the cytokines do not show significant level changes, the tendency was that cytokine levels are more likely to change under the HIIT than hemolytic parameters.

The use of the HIIT program not only in PD but also in other neurodegenerative diseases can bring many positive effects. The research presented in this paper shows that the reduction of inflammatory processes with a simultaneous increase in anti-inflammatory processes and the long-term persistence of changes induced by HIIT may contribute to the improvement of the quality of life of people with chronic diseases in which inflammation plays an important role. Systematic exercise and the maintenance of a high level of intensity can be considered as a good supportive non-pharmacological treatment option for the patients and effectively contribute to slowing down the progression of the disease.

## Data Availability

The datasets generated during and/or analyzed during the current study are available from the corresponding author upon reasonable request.
