# Correction: High-intensity interval training modulates inflammatory response in Parkinson’s disease

**DOI:** 10.1007/s40520-022-02208-7

**Published:** 2022-08-26

**Authors:** Paulina Malczynska-Sims, Małgorzata Chalimoniuk, Zbigniew Wronski, Jaroslaw Marusiak, Anna Sulek

**Affiliations:** 1 Department of Genetics, Institute of Psychiatry and Neurology, 9 Sobieskiego St, 02-957 Warsaw, Poland; 2 Department of Physical Education and Health in Biała Podlaska, Józef Piłsudski University of Physical Education in Warsaw, 2 Akademicka St, 21-500 Biała Podlaska, Poland; 3 Department of Rehabilitation, Medical Faculty, Medical University of Warsaw, 61 Żwirki i Wigury St., 02-091 Warsaw, Poland; 4 Department of Kinesiology, University School of Physical Education in Wroclaw, Wroclaw, Poland

## Correction to: Aging Clinical and Experimental Research 10.1007/s40520-022-02153-5

In the original version of this article, the given and family names of Paulina Malczynska-Sims, Małgorzata Chalimoniuk, Zbigniew Wronski, Jaroslaw Marusiak, Anna Sulek have been incorrectly published. Now they have been corrected.

The original article has been updated.

